# An integrative model for recurrence in ovarian cancer

**DOI:** 10.1186/1476-4598-7-8

**Published:** 2008-01-22

**Authors:** Alexandros Laios, Sharon A O'Toole, Richard Flavin, Cara Martin, Martina Ring, Noreen Gleeson, Tom D'Arcy, Eamonn PJ McGuinness, Orla Sheils, Brian L Sheppard, John J O' Leary

**Affiliations:** 1 Department of Obstetrics and Gynaecology, Trinity College Dublin, Trinity Centre for Health Sciences, St. James's Hospital, Dublin 8, Ireland; 2 Department of Histopathology, Trinity College Dublin, Trinity Centre for Health Sciences, St James's Hospital, Dublin 8, Ireland

## Background

Ovarian cancer is one of the commonest cancers in women and the leading cause of death from gynaecological malignancy in the western world. About 205,000 cases of ovarian cancer are diagnosed worldwide each year [1]. It accounts for 3% of female cancers in Ireland with over 350 new cases each year [2]. Marked heterogeneity is a hallmark of the disease, not only in tumor histotype and grade but also in response to chemotherapy and overall prognosis [3]. Over 90% of cases arise from the surface epithelium. Serous adenocarcinomas are the commonest and account for 40%–50% of malignant neoplasms [4].

The majority of ovarian cancers present in advanced stages (III or IV) and are treated by surgery and systemic chemotherapy, most frequently carboplatin and paclitaxel. Conventional chemotherapy is still unsatisfactory as it ignores aspects of tumor biology. Despite an initial 70–80% response rate, current therapy is frequently followed by recurrence which is often resistant to chemotherapy, as demonstrated by the 5–20% long-term survivors [5]. Understanding the biological mechanisms underlying recurrence of ovarian cancer and addressing chemoresistance is of the utmost importance for improving treatment and outcome of the disease. Previous studies using single gene biomarkers to predict tumor response have been inconclusive. Patterns of gene expression for recurrence are likely to involve multiple gene pathways and integration of these pathways.

High throughput discovery tools such as DNA microarrays have enabled the study of gene expression profiles of large numbers of cancer samples. Several groups have successfully applied expression array technology to the molecular classification of ovarian cancers [6,7], confirming a process of differentiation in the progression of ovarian cancer [8] and others have attempted to predict outcome and chemotherapeutic response [9,10]. Previous studies in our laboratory focused on assays and markers to predict response to chemotherapy [11,12].

While numerous studies have characterized primary ovarian cancers, less information is available regarding expression patterns of recurrent ovarian cancers. The aim of this study was to determine whether primary and recurrent ovarian tumors could be distinguished based on their gene expression profiles, by using gene expression arrays and to identify potential biomarkers of recurrence.

## Results

### Gene expression profiling distinguishes primary and recurrent ovarian cancers

**Figure 1:**
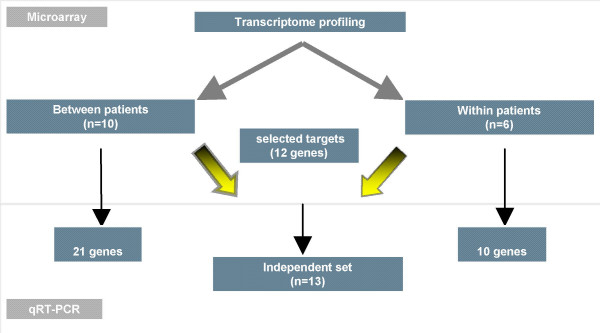
Flow chart of our study design. 2 cohorts were used in this study: In the first one, we selected a homogeneous series of primary and recurrent serous papillary adenocarcinomas from different patients(Between patient cohort). The second cohort consisted of 3 paired ovarian cancers (primary and recurrent samples coming from the same patient) but of different histology (Within patient cohort). Selected genes identified from microarray experiments were validated for both cohorts and a subset of these genes (n = 12) were validated in an independent set (test set) of 13 serous papillary adenocarcinomas using TaqMan^® ^PCR.

A flow chart of our study design is shown in Figure 1. To identify potentially important mediators of recurrence in the most frequent histological pattern of ovarian cancer, we performed cDNA microarray experiments on a homogenous set of primary and recurrent serous papillary ovarian tumors (cohort 1). Gene expression profiling revealed a total of 907 genes as differentially expressed between primary and recurrent samples at p < 0.01. Using the more stringent false discovery rate (FDR 0.1), this list was narrowed down to 182 genes. Included in this FDR list (with the exception of *CLDN16*, which was the top differentially expressed gene in the p value list), were *BTC*, *S100B*, *IL27RA*, *CSRP2*, *ARFRP1*, *PVRL2*, *WASF1*, *STARD10*, *LASS4*, *LGALS3BP*, *CASK*, *IFNGR1*, *PGM2L1*, *USF2*, *PERP*, *ESM*, *CHORDC1*, *RNPC1*, *MGAT4B and CACNA1D *which were selected for validation. Table 1 displays the fold changes observed for these genes, the corresponding probe identification and the p values.

**Table 1:** List of gene targets selected for TaqMan^® ^validation in cohort 1

**Gene Symbol**	**Probe ID**	**P value**	**Array fold change**	**Expression in recurrent vs primary**	**Assay ID**
*CLDN16*	163630	0.0068	9	Upregulated	Hs00198134_m1
*S100B*	114188	0.0001	6.12	Upregulated	Hs00389217_m1
*CACNAID*	127127	0.00012	3.9	Upregulated	Hs00167753_m1
*BTC*	112710	0.0006	2.67	Upregulated	Hs00156140_m1
*IL27RA*	145754	0.0005	1.82	Upregulated	Hs00175472_m1
*CHORDC1*	183675	0.0002	1.359	Upregulated	Hs00854389_m1
*LASS4*	155606	1.78E-05	9.02	Downregulated	Hs00226114_m1
*STARD10*	115998	4.26E-05	6.03	Downregulated	Hs00246405_m1
*CSRP2*	137165	0.0003	5.7	Downregulated	Hs00426717_m1
*RNPC1*	215065	0.0003	4.87	Downregulated	Hs00246405_m1
*ARFRP1*	118657	0.0003	4.85	Downregulated	Hs00182389_m1
*ESM1*	174810	0.005	4.68	Downregulated	Hs00199831_m1
*CASK*	143978	0.00035	4.42	Downregulated	Hs00177620_m1
*WASF1*	118398	0.0001	4.32	Downregulated	Hs00187514_m1
*PVRL2*	128369	0.0002	4.23	Downregulated	Hs00161054_m1
*MGAT4B*	186405	0.0002	4.13	Downregulated	Hs00365001_m1
*LGALS3BP*	185853	9.49E-05	4.12	Downregulated	Hs00174774_m1
*PERP*	141176	0.0004	3.52	Downregulated	Hs00751717_m1
*PGM2L1*	138177	0.0005	3.04	Downregulated	Hs00328100_m1
*IFNGR1*	190183	0.0003	2.57	Downregulated	Hs00166223_m1
*USF2*	144617	0.0005	1.94	Downregulated	Hs00231528_m1

To address whether recurrence follows similar patterns and to avoid individual genetic variation, we profiled paired samples from the same patient (cohort 2). A total of 586 genes were differentially expressed between primary and recurrent at p < 0.05 and this was reduced to 75 genes at p < 0.01. Genes with a fold change >4, upregulated in recurrent compared to primary tumors included: *Septin 6*, *ZNF218*, *S100A8*, *MMP9*, *FOXF1 *and *ILIR2 *(Table 2).

**Table 2:** List of gene targets selected for TaqMan^® ^Validation in cohort 2

**Gene Symbol**	**Probe ID**	**P value**	**Array fold change**	**Expression in recurrent vs primary**	**Assay ID**
*S100A8*	196494	0.034	11.6	Upregulated	Hs00374263_m1
*ZNF218*	217273	0.002	6.06	Upregulated	Hs00542836_m1
*MMP9*	112640	0.0169	4.975	Upregulated	Hs00234579_m1
*IL1R2*	186009	0.038	4.46	Upregulated	Hs00174759_m1
*SEPTIN6*	122775	0.019	4.16	Upregulated	Hs00248408_m1
*NRG2*	170215	0.013	3.6	Upregulated	Hs00171706_m1
*SPDEF*	155169	0.005	2.87	Upregulated	Hs00171942_m1
*TJP3*	182089	0.013	2.7	Upregulated	Hs00274276_m1
*FGF2*	113042	0.03	2.7	Upregulated	Hs00266645_m1
*FOXF1*	145754	0.0015	6.58	Downregulated	Hs00230962_m1

**Figure 2:**
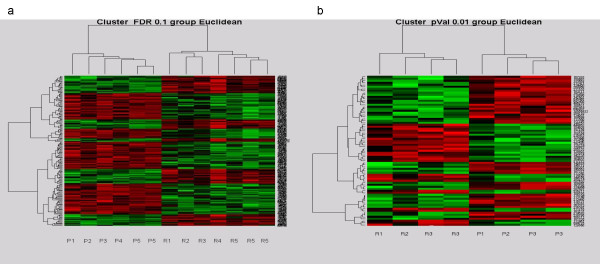
Hierarchical cluster heatmaps demonstrating distinct patterns of gene expression between primary and recurrent ovarian tumors. (a) Heatmap of the ovarian tumors in cohort 1 based on the FDR0.1 list with the primary clustering on the left and the recurrent samples on the right. Vertical bars represent the samples and the horizontal bars represent the genes. Green bars reflect downregulated genes and red bars upregulated genes. (b) Heat map discriminating recurrent (left) and primary (right) ovarian tumours in cohort 2 based on the p0.01 list. P, primary tumours; R, recurrent tumors.

Hierarchical heat maps, presented in Figures 2a and 2b, for both cohorts, demonstrated distinct gene expression patterns between primary and recurrent ovarian cancers.

**Figure 3:**
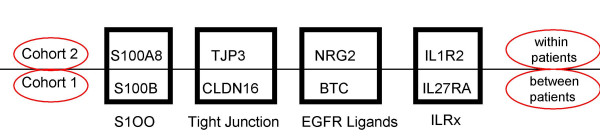
Gene families involved in the molecular regulation of recurrence in ovarian cancer. Some of the upregulated genes in recurrent compared to primary ovarian carcinomas that we validated in cohort 2 belong in the same gene families with some of the upregulated genes validated in cohort 1. Upregulation of tight junction proteins and EGFR ligands, development of a cytokine response via interleukin receptors and intracellular signaling via calcium binding S100 proteins seem to contribute to the "recurrent" signature and possibly have a role in drug resistance.

Notably, upregulated genes in the recurrent compared to primary tumors in cohort 1 and 2 segregated in the same gene families. Included among these genes are *S100B *and *S100A8 *belonging to the S100 family of calcium binding cytoplasmic proteins, *TJP3 *and *CLDN16 *belonging to the family of tight junction proteins, *BTC *and *NRG2 *belonging to the family of EGFR ligands, and interleukin receptors *IL1R2 *and *IL27RA *(Figure 3).

### Validation of gene expression by qRT-PCR

**Figure 4:**
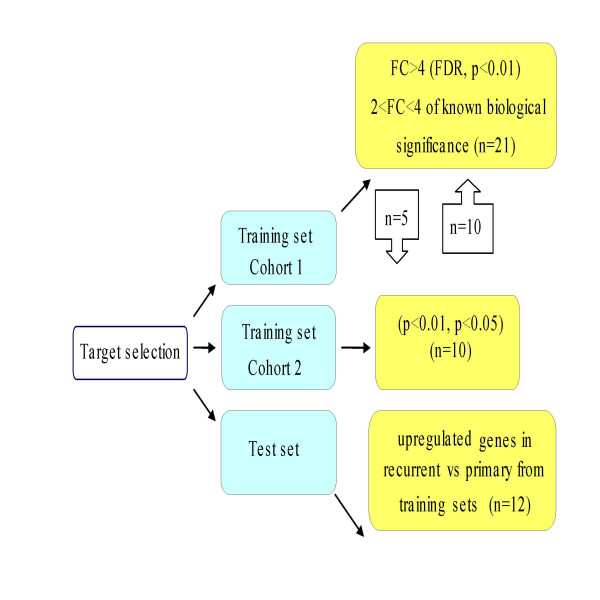
TaqMan^® ^PCR validation of target genes identified in both training and test sets. Gene selection for TaqMan^® ^validation was based on the most differentially expressed genes from the p and FDR value list with a fold change > 4 but also included genes that had a 2–4 fold change and also some genes involved in the most differentially expressed pathways. Priority was given to selection of genes upregulated in recurrent compared to primary samples, which might provide "recurrence" signatures in ovarian cancer. Upregulated genes validated in both cohorts were alternatively interrogated (external validation) and further advanced for validation in the test set. Independent validation on a test set refers to completely distinct samples of serous histology that were not previously employed in marker development (n = number of gene targets selected for validation).

**Figure 5:**
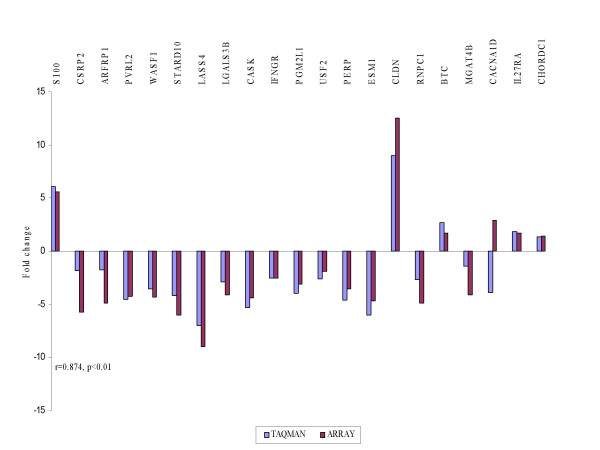
TaqMan^® ^PCR validation of microarray experiments in cohort 1. The fold changes in the arrays were plotted against the relative quantitation from TaqMan^® ^in recurrent vs primary tumours. The TaqMan^® ^values are displayed in blue and the array results in red. Spearman correlation r showed high concordance between the 2 experiments.

**Figure 6:**
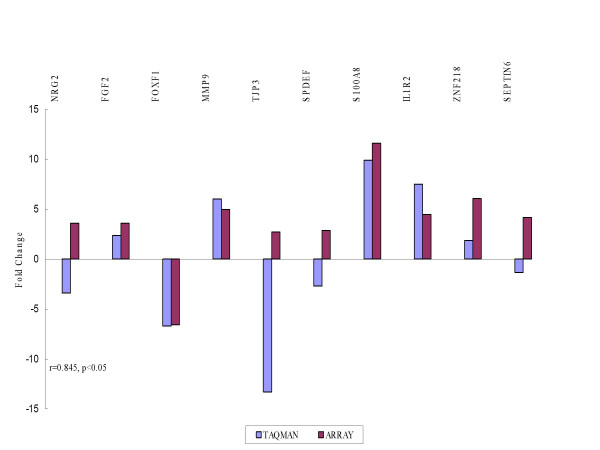
TaqMan^® ^PCR validation of microarray experiments in cohort 2. A similar concordance was observed as in cohort 1.

Using TaqMan^® ^PCR we validated dysregulated genes against different interrogation sets in order to select those likely to represent markers of recurrence (Figure 4). A list of the genes chosen for validation together with their molecular function and biological processes is shown in additional file 1. Correlation was carried out using Spearman correlation co-efficient. The fold changes in the arrays were plotted against relative quantitation from the TaqMan^® ^analyses of recurrent versus primary tumors. High concordance was revealed between TaqMan^® ^and microarray experiments in cohort 1 (r = 0.874, p < 0.01) (Figure 5) and cohort 2 (r = 0.845, p < 0.05) (Figure 6).

**Figure 7:**
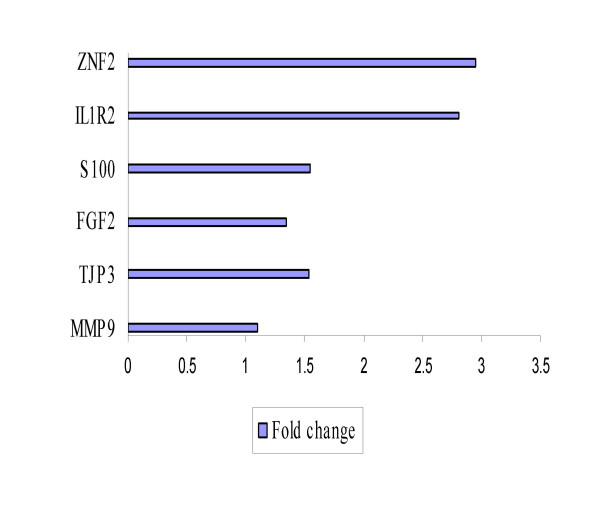
External validation of a subset of upregulated genes in cohort 2 that validated in cohort 1. Bars indicate the relative overexpression of target genes in recurrent vs primary tumors. IL1R2 and ZNF218 gave the best distinction between recurrent and primary tumors with greater than twofold changes.

*IL1R2 *and *ZNF218 *identified in cohort 2 as upregulated in recurrent, when validated in samples from cohort 1, gave the best distinction with fold changes of 2.81 and 2.94 respectively (Figure 7). No significant difference was observed between recurrent and primary samples for the remaining 13 which is in accordance with the array results.

**Figure 8:**
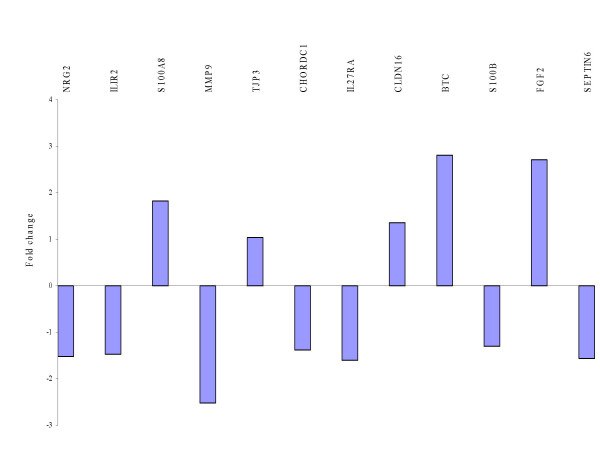
Independent TaqMan^® ^PCR validation of a set of selected genes from both cohorts in a test set of serous papillary adenocarcinomas of varying grade and stage. BTC and FGF2 provided the best distinction between recurrent and primary tumours with fold changes of 2.8 and 2.71 respectively.

Consecutively independent validation of a subset of the above genes (n = 12) from both cohorts was carried out in our test set of primary and recurrent serous papillary adenocarcinomas (n = 13) using TaqMan^® ^PCR to identify if these targets were possible markers of recurrence for serous papillary adenocarcinomas (Figure 8). *BTC *and *FGF2 *provided the best distinction between recurrent and primary tumors with fold changes of 2.8 and 2.71 respectively. Adding 2 recurrent samples of different histologies to the previously homogeneous histological sample cohort conferred no statistical significance for any of the validated genes (fold changes < 2). These were subsequently excluded from the analysis to preserve homogeneity in the test set.

The results confirm the utility of the derived set of markers as potential markers of recurrence. Recurrence is rather multifactorial as indicated by non identification of a single biochemical pathway to relate the above targets using the ingenuity program [13].

## Discussion

This investigation demonstrates a distinct pattern of gene expression between primary and recurrent ovarian carcinomas in vivo and represents an effort to discover potentially important mediators of recurrence. It also addresses whether mechanisms of gene dysregulation in primary versus recurrent paired tumors are unique to those pairs. In order to address chemoresistance and minimise parameters introducing variations, well characterized tumors of the commonest histological subtype (serous papillary adenocarcinoma) were selected in cohort 1. In cohort 2, we used paired samples with identical genetic background (from the same patients). Such samples are valuable since second-look laparotomy in Europe is a rather uncommon practice in the treatment of ovarian cancer. To our knowledge, this dual approach of profiling primary and recurrent ovarian cancers in vivo has never been previously performed.

Unsupervised hierarchical clustering of the samples in both cohorts unambiguously separated primary and recurrent tumors. The substantial number of gene expression differences between the two groups, in particular cohort 2, reflects progression of the tumours from a primary to a relapsed state, but is also consistent with the hypothesis that tumor cells surviving chemotherapeutic treatment alter their gene expression allowing them to withstand the selective pressure of the drugs used. In fact, the "log kill" effect of cytotoxic chemotherapy significantly reduces tumor cells that are sensitive to the administered therapy [14]. Hence, one is inclined to think that recurrent tumors are enriched with resistant clones and likely to display molecular signatures more associated with acquired chemoresistance. Acquired resistance is likely to reflect changes in gene regulation rather than mutation-dependent clone selection [15], especially in solid tumors which have relatively low doubling rates.

Nonetheless, genes upregulated in recurrent compared to primary advanced ovarian tumors could be invoked as "recurrent metastasis virulent genes" that provide a selective advantage in recurrent sites but not in primary tumorigenicity [16].

Validation of targets was satisfactory as indicated by Spearman coefficients. Some variation may be due to differences in sensitivity between the two techniques, probably because of the number of absent cells made during normalisation of the microarray data or the asymmetry in the number of samples used in both cohorts.

We identified and validated genes upregulated in the recurrent tumors, which may be the signature expression pattern of drug resistant cancers (Table 1). Some of the genes identified in the two cohorts belong to the same gene families corroborating the importance of several distinct gene families in the molecular regulation of ovarian cancer recurrence. Since all patients in both cohorts were treated with platinum based therapy and some of the identified genes are thought to be correlated to the mode of action of chemotherapeutic agents as discussed below, it is interesting to speculate that some of the mechanisms involved in recurrence are specific to the drugs used. Whether the genes represent etiologic causes of drug resistance or treatment failure remains to be further answered.

*S100B *was the most differentially upregulated gene in recurrent serous tumors (FDR 0.1). It constitutes a calcium binding cytoplasmic protein, involved in intracellular signaling. Historically used in the clinical management of malignant melanomas, it was found to be a negative regulator of p53 [17]. If elevated S100B levels cause downregulation of p53, apoptotic pathways are not induced resulting in uncontrolled tumor growth or resistant phenotypes [18]. A recent proteomic study identified a putative S100 protein to be upregulated in cisplatin resistant cell lines [19]. Other members of the S100 family, including *S100A8*, identified in cohort 2, are overexpressed in common cancers. S100B is also involved in the assembly and disassembly of microtubules[20] and therefore might interfere with the mechanism of action of taxanes.

*CLDN16 *was the most upregulated gene in the recurrent serous tumors (p < 0.01) in cohort 1. It belongs to the family of claudins, tight junction (TJs) – associated proteins endowed with well-characterized roles in individual viability, epithelial differentiation and tumor growth probably by stabilizing tumor cell connection. A link between cisplatin exposure and cytoskeletal alterations has been reported [21]. Notably, *CLDN16 *has been characterized as a novel human ovarian cancer-specific transcript using serial analysis of gene expression data [22]. Other members of the family, *CLDN3 *and *CLDN4 *are frequently overexpressed in ovarian cancer. They have also been described to function as receptors for Clostridium Perfringens Enterotoxin [23], a finding that might open novel treatment approaches in ovarian cancer. Expression levels of *CLDN10 *are associated with recurrence of primary hepatocellular carcinoma [24]. A proteomic study identified *CLDN4 *as overexpressed in cisplatin resistance cell lines [19]. *TJP3*, another discriminatory gene identified in cohort 2, anchors to the cytoplasmic tail of claudins [25]. No studies on the relationship between TJs and chemotherapy response have been carried out. The functional role of *CLDN16 *and *TJP3 *in carcinogenesis is unknown. As loss of cell-cell contact has been noted to induce apoptosis [26], one would hypothesise that increased contact through tight junctions as a result of upregulation of these component proteins may favor tumor survival. Transmembrane proteins are also more likely to remodel the tumor microenvironment to favor drug resistance [27].

Of particular interest are putative markers among the coexpressed genes *ILIR2 *and *ZNF218 *that we validated in cohort 2 and they provided the best discrimination between primary and recurrent serous neoplasms in cohort 1. The role of cytokines and their receptors is well established in the epithelial cancer microenvironment [28] with *IL8 *and *IL6 *known to be involved ovarian cancer pathogenesis [29]. A novel regimen of immunomodulatory cytokines and carboplatin show promising results on completion of a phase II clinical trial, in recurrent ovarian cancer [30]. *IL-1*, a proinflammatory cytokine, is required for tumor invasiveness and angiogenesis in a variety of malignant lesions [31] and particularly in metastatic human tumor specimens [32]. Targeting *IL1R2 *may be an appropriate therapeutic strategy for inhibiting tumor angiogenesis. *IL1R2 *may provide an insight into the biology of recurrent ovarian tumors, suggesting an initial immune response to the relapsing neoplasm and may secondly represent a surrogate marker of recurrence in ovarian cancer.

*ZNF 218 *encodes for a newly described zinc finger protein located on chromosome 20q13.2 and has never been previously reported in ovarian cancer. Frequent amplification of DNA at 20q has been demonstrated by comparative genomic hybridization in ovarian cancer [33] and is associated with poor prognosis [34]. Given that amplification appears to be the predominant mechanism leading to overexpression of genes, *ZNF 218 *emerges as a strong candidate oncogene related to ovarian cancer [35].

External validation using independent sets of samples is a critical step in view of the potential for false-discovery using microarrays. In this regard, a test set of 13 serous papillary ovarian adenocarcinomas (primary and recurrent) of various stages and grades, given the same initial chemotherapy treatment, was used for relative quantitation of 12 targets upregulated in recurrent versus primary tumors. An elevated mRNA expression (FC > 2) was observed for 2 out of 12 genes, namely *BTC *and *FGF2*, which bind to Epidermal Growth Factor Receptor (EGFR or ErbB1) and Fibroblast Growth Factor Receptor (FGFR) respectively. These genes gave the best distinction between primary and recurrent tumors in our test set of serous tumors. The inclusion of two tumors (n = 2) of nonserous histologies (i.e. yolk sac and clear cell) in the previously homogenous test set conferred no statistical significance for any of the validated genes (FC < 2), suggesting that various histological types may have variable chemosensitivities. Since EGFR and FGFR stimulate a similar repertoire of intracellular signaling pathways [36], this certain pattern of expression evident in the recurrent serous ovarian cancers deserves special consideration.

Despite the well established significance of EGFR in the progression of ovarian cancer [37], the role of EGF ligands in ovarian cancer is still poorly understood. Overexpression of EGFR is found in up to 75% of ovarian cancers and is associated with chemoresistance and poor prognosis [38]. Our study suggests that overproduction of ligands (*BTC *and *NRG2*), rather than overproduction of receptors could be the predominant mechanism that the ErbB pathway uses to generate cancer cell proliferation signals. Combined targeting of ErbB receptors and their ligands produces a synergistic antitumor effect [39] because of their non-overlapping functions.

*BTC *binding to EGFR initiates significant signal transduction pathways, such as MAPK and PI-3-kinase-Akt (PI3K/Akt) [40] in a metalloprotease dependent manner [41,42]. The PI3K/Akt pathway promotes cell survival and has been identified as a potential contributor to drug resistance [43]. *MMP-9 *co-upregulation was also observed in our study, which is in agreement with the finding that *MMP-9 *(and *MMP-14*) mRNA levels are selectively increased in response to EGFR activity in ovarian tumor cells [44]. Metalloproteases have also been described in a novel drug-resistant phenomenon [45]. EGFR ligands and matrix metalloproteases constitute a vascular-remodeling program that facilitates pathological angiogenesis in mammary tumors [46].

FGF ligands are mitogenic growth factors, generally known to act in a local manner. A recent study on the repertoire of mutated human cancer genes, based on the family of protein kinases, identified the FGF signaling pathway to confer growth advantage by the highest enrichment for kinases containing "driver" mutations [47]. *FGF2 *has been described in prostate cancer [48] and implicated in cancer invasion and metastasis, probably through upregulation of *MMP9 *[49], which was also upregulated in our study, as mentioned above. *FGF2 *critically modulates mesenchymal-to-epithelial transition, which is now widely accepted to contribute to carcinoma invasiveness [50]. These findings would explain the acquired metastatic capacity or recurrence pattern of advanced epithelial ovarian cancers, which are mesothelially derived and suggest a role for *FGF2 *as an epithelial marker in strategies to block epithelialization of metastases.

## Conclusion

**Figure 9:**
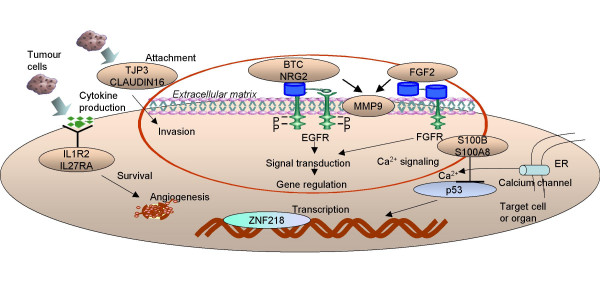
An integrative model for recurrence in ovarian cancer. Schematic representation of putative genes and gene families involved in the recurrence of ovarian carcinomas. According to our current working concept, tumour cells during relapse produce adhesion molecules to mediate attachment and invasion via co-overexpression of matrix metalloproteinases, cytokines and inflammatory mediators to stimulate survival and a variety of growth factors bound to their cognate receptors to fully proliferate in order to confront and modulate their immediate environment, which they must eventually overtake.

Collectively, our data propose an integrative model for recurrence in ovarian cancer, in which tumor cells during relapse produce adhesion molecules to mediate attachment, cytokines and inflammatory mediators to stimulate survival and a variety of growth factors bound to their cognate receptors to fully proliferate in order to confront and modulate their immediate environment, which they must eventually overtake (Figure 9).

To date, the histological type of the ovarian cancer is not used as a factor to determine chemotherapy management. As the best strategy for second-line chemotherapy has not yet been defined, the proposed panel of genes could reduce at least in individual patients, unnecessary chemotherapy treatment and toxicity or alternatively could be readily used for early detection of disease recurrence. Clearly, a definite role for the candidate targets will be provided by functionally investigating the mechanisms involved in the development of recurrence and drug resistance. These types of analyses may lead to novel approaches for the development of therapy aimed at reversing or decreasing drug resistance or alternatively optimization of the already available standard drugs [51]. A synchronous targeting of co-amplified genes may in the future offer better treatment for recurrent ovarian cancer.

## Materials and methods

### Patients and tissue samples

The study consisted of 2 cohorts: cohort 1 comprised 5 primary serous papillary adenocarcinomas, grade 3, FIGO stage III and 5 recurrent serous papillary adenocarcinomas of the same grade. The mean age in years for patients in the primary and the recurrent group in cohort 1 was 62.6 (range 48–84) and 52.4 (range 43–68). The recurrent group consisted of patients for which primary surgery was performed prior to the commencement of the study. In cohort 2, 3 paired ovarian cancers were used (primary and recurrent coming from the same patient), but of different histology, namely papillary serous, mixed mullerian and clear cell carcinomas. The mean age in years for patients in cohort 2 was 52 (range 39–74) for the primary and 53.3 (range 43–74) for the recurrent.

To further validate identified gene targets in cohorts 1 and 2, an independent set (test set) of serous papillary ovarian adenocarcinomas were interrogated. This additional cohort comprised of 13 serous papillary ovarian adenocarcinomas, 10 primary and 3 recurrent cases. An additional two patients with different histological subtypes of recurrent ovarian cancer (one patient with well differentiated clear cell carcinoma and one patient with dedifferentiated germ cell carcinoma) were also included in the test set.

All tumors were staged according to the International Federation of Gynaecology and Obstetrics standards (FIGO). Patients were optimally debulked (residual disease of <1 cm in greatest diameter), had received no neoadjuvant treatment before surgery and they were all treated post operatively with paclitaxel and platinum. The two additional recurrent patients in the test set were optimally debulked, one received postoperative carboplatin/paclitaxel and the other bleomycin/etoposide/platinum.

All samples were removed as part of patient treatment for ovarian cancer at St James's Hospital, Dublin, Ireland. The study had approval of the hospital ethics committee and informed consent was obtained from each patient by the research team prior to surgery.

Specimens were snap frozen on collection within 1 hour of surgery and stored at -80°C. After tissue processing in a cryostat at -20°C, frozen sections were cut and mounted on slides. The slides were stained with H&E and examined by a pathologist to ensure >70% presence of tumor cells.

### cDNA Arrays

Samples were placed in liquid nitrogen, ground thoroughly with a mortar and pestle and homogenized in RLT buffer (Qiagen Ltd, UK). Total RNA was extracted using the Qiagen RNAeasy (Hilden, Germany) RNA Mini Kit and on-column RNase-free DNase digestion was performed according to the manufacturer's instructions. RNA quantity and quality was determined using Nanodrop spectrophotometer (Nanodrop Tehnologies) and the Agilent 2100 Bioanalyzer (Agilent Technologies, Waldbronn, Germany).

Gene expression profiles were examined using the Applied Biosystems (ABI) (Foster City, CA, USA) Human Genome Survey Microarrays V2.0 system. These arrays contain 32,878 oligonucleotide probes (60 base pairs long) and target a complete annotated and fully curated set of 29,098 human genes from the public and Celera databases. Double stranded cDNA was prepared from 2 micrograms of total RNA using an oligo dT priming approach followed by in vitro transcription and labelling to generate Digoxigenin (DIG) labelled cRNA (ABI Chemiluminescent RT-IVT Labelling Kit) according to the manufacturer's protocol.

Each microarray was first pre-hybridized at 55°C for 1 hr in hybridisation buffer with blocking reagent. 10 μg of labelled cRNA targets were randomly fragmented by incubating with fragmentation buffer at 60°C for 30 min, mixed with internal control target (ICT, 24-mer oligo labelled with LIZ fluorescent dye) and then hybridized to each pre-hybridized microarray at 55°C for 16 hr. Following hybridisation, the arrays were washed with hybridisation wash buffer and chemiluminescence rinse buffer and stained with anti-DIG alkaline phosphatase, further enhanced with Chemiluminescence Enhancing Solution and finally with Chemiluminescence Substrate. Each array was then scanned on the ABI 1700 Platform for image collection. Technical replicates were performed on a subset of samples to ensure concordance and only those with greater than 90% concordance were considered valid.

### Bioinformatics

The R statistical package, a free language and environment was developed to work with the Applied Biosystems whole genome microarray platform for all the analysis (R Development Core Team, 2004) [52]. Following quality control assessments, a two-step algorithm automatically processed raw image data, including gridding and quantification so that genes were deemed undetectable if they had signal-to-noise (S/N) ratio threshold > 3 in 75% of the samples. Data were normalized with quantile normalization. Paired t and ANOVA tests were performed to generate p values for statistical differences between the two groups, with p < 0.05 considered significant. The p values were further adjusted using more stringent Benjamini-Hochberg false discovery rate (FDR) at preset levels: 0.01, 0.1. Gene expression profiles were examined based on fold change, p and FDR values. The R package was used to visualize hierarchical clustering between differentially expressed genes. Unsupervised hierarchical clustering was applied to the data set using the Unweighted Pair Group Method with Arithmetic Mean (UPGMA) based on Euclidean distance as the similarity measure. Functional classification of the data and gene ontology was defined by using the PANTHER (Protein Analysis THrough Evolutionary Relationships) Classification System. A binomial statistical tool was employed to compare gene lists to a reference list (i.e. the complete human genome) to determine over- or under-representation of PANTHER classification categories. To further refine the gene lists, it was investigated if any of these genes were known to interact biologically. To this end, pathway analysis using the Ingenuity Pathways Analysis (IPA) tool was used which is a web-based software application that enables scientists to identify the biological mechanisms, pathways and functions most relevant to their experimental datasets or genes of interest [13].

Expression data from microarray and TaqMan^® ^PCR data were correlated with nonparametric Spearman's correlation coefficient (r) to avoid distributional assumptions.

### Validation of gene expression targets

RNA was initially reverse transcribed using a High Capacity cDNA Archive Kit (Applied Biosystems, CA, USA) and then was amplified in a 10 μL PCR reaction according to the manufacturer's recommended protocol and amplification steps: denaturation at 95°C, followed by 40 cycles of denaturation at 95°C for 15 sec and then annealing at 60°C for 1 min. All reactions were carried out on the ABI Prism 7000 Sequence detection system (Applied Biosystems, Applera UK, Cheshire, UK) using the TaqMan^® ^Universal PCR master Mix and Assays on demand (Applied Biosystems). Gene symbols and assay ID's are shown in Table 1. Relative quantitation was carried out using the "Delta-Delta Ct" (ΔΔCt) method with 18S ribosomal RNA as an endogenous control. Transcript quantification was performed in triplicate for each sample.

The mRNA levels of 31 overall differentially expressed genes were validated using the assay on Demands™ gene expression products (Applied Biosystems) real-time quantitative RT-PCR assay (TaqMan^® ^Gene expression assay) with an ABI PRISM 7000 sequence detection system (Applied Biosystems, CA, USA) (Table 1).

21 genes were selected for our validation in cohort 1. We selected some of the most differentially expressed genes (FC > 4) but we also included genes that had a 2–4 fold change and also some genes involved in the most differentially expressed pathways using the PANTHER binomial statistics tool.

In cohort 2, 10 targets were selected for validation. These included some of the most differentially expressed genes from both p value lists but also genes of known biological significance.

### External and independent test set validation

Randomly selected genes identified in cohort 1 as upregulated in recurrent compared to primary samples (n = 5) were validated against cohort 2 samples. Likewise, randomly selected genes identified in cohort 2 with a special emphasis on those again upregulated in recurrent versus primary (n = 10) were tested against cohort 1 samples.

Validation of 12 upregulated genes in the recurrent samples from both cohorts was performed in an independent set of 10 primary and 3 recurrent serous papillary adenocarcinomas. An additional 2 recurrent samples of different histology were included.

## Competing interests

The author(s) declare that they have no competing interests.

## Supplementary Material

Additional file 1 List of selected genes differentially expressed in recurrent versus primary tumours in cohorts 1 and 2. The list includes selected genes from both cohorts that we chose to validate using TaqMan and gives information about their molecular function, biological processes or pathways involved. Click here for file
