# Supplementary material for: An integrative model for recurrence in ovarian cancer
Source: Mol Cancer. 2008 Jan 22;7:8. doi: 10.1186/1476-4598-7-8 (PMC2248209; doi:10.1186/1476-4598-7-8)
Supplement: Additional file 1 — List of selected genes differentially expressed in recurrent versus primary tumours in cohorts 1 and 2. The list includes selected genes from both cohorts that we chose to validate using TaqMan and gives information about their molecular function, biological processes or pathways involved. [file 1476-4598-7-8-S1.doc]

**Additional file 1** List of selected genes differentially expressed in recurrent versus primary tumours in cohorts 1 and 2 including information about their molecular function, biological processes or pathways involved. Some of these genes have not been previously implicated in ovarian cancer. These genes were chosen to be validated as they might represent markers of recurrence*.*

**Gene Symbol Molecular function Biological processes/pathways**

*Cohort 1*

CLAUDIN 16 Tight junction Cell structure

S100B Ca2+ related protein Assembly of microtubules and

intermediate filaments

CACNAID Voltage gated Ca2+ protein Transfer of cations, muscle

contraction

BTC EGFR binding ligand EGFR signaling

IL27RA Cytokine receptor Inflammation, immunity

CHORDC1 Cysteine and histidine rich Transport signaling

zinc binding protein

LASS4 Ceramide synthesis Lipid metabolism

STARD10 Transfer protein Lipid and fatty acid transfer

CSRP2 Actin binding protein Cell growth

RNPC1 Transcription factor RNA Nucleic acid metabolism

binding protein

ARFRP1 Small GTPase Cadherin signaling

WASF1 Rho GTPase Link between TK receptors and

cytoskeleton

PVRL2 Actin binding protein Defense immunity,

Receptor Signal transduction

MGAT4B Glycosyl transferase Protein glycosylation, Cell growth

LGALS3BP Galectin binding protein Cell adhesion mediating signaling

PERP P53 induced protein related Apoptosis

PGM2L1 Mutase O antigen synthesis

IFNGR1 Receptor Interferon signalling pathway

USF2 Transcription factor Regulation of steroid, nucleic acid and lipid metabolism

CASK Protein kinase Protein phosphorylation, cell

communication

ESM1 Endothelial cell specific Cell structure

growth factor

*Cohort 2*

S1OOA8 Ca2+ related protein Assembly of microtubules and

intermediate filaments

ZNF218 Transcription factor Transcription regulation

MMP9 Extracellular matrix protein Proteolysis

IL1R2 Cytokine receptor Inflammation, immunity

SEPTIN6 Cyoskeletal protein Cytokinesis

NRG2 ERbB receptor binding ERbB receptor signalling

ligand pathway

SPDEF ETS containing Transcription regulation

transcription factor

TJP3 Tight junction Cell structure

FGF2 FGF binding ligand FGF signaling pathway

*FOXF1* Transcription factor Cell cycle control, proliferation, differentiation
